# Polymorphisms in Genes Involved in Fatty Acid β-Oxidation Interact with Dietary Fat Intakes to Modulate the Plasma TG Response to a Fish Oil Supplementation

**DOI:** 10.3390/nu6031145

**Published:** 2014-03-18

**Authors:** Annie Bouchard-Mercier, Iwona Rudkowska, Simone Lemieux, Patrick Couture, Marie-Claude Vohl

**Affiliations:** 1 Institute of Nutrition and Functional Foods (INAF), Laval University, 2440 Hochelaga Blvd., Quebec, QC, G1V 0A6, Canada; E-Mails: annie.bouchard-mercier@fsaa.ulaval.ca (A.B.-M.); simone.lemieux@fsaa.ulaval.ca (S.L.); patrick.couture@crchul.ulaval.ca (P.C.); 2 Department of Food Sciences and Nutrition, Laval University, 2425 de l’Agriculture St., Quebec, QC, G1K 7P4, Canada; 3 Endocrinology and Nephrology, CHU de Quebec Research Center, 2705 Laurier Blvd., Quebec, QC, G1V 4G2, Canada; E-Mail: iwona.rudkowska@kin.ulaval.ca

**Keywords:** gene-diet interaction, omega-3 polyunsaturated fatty acid, fish oil, fatty acid β-oxidation, single nucleotide polymorphism, triglyceride

## Abstract

A large inter-individual variability in the plasma triglyceride (TG) response to an omega-3 polyunsaturated fatty acid (*n*-3 PUFA) supplementation has been observed. The objective was to examine gene-diet interaction effects on the plasma TG response after a fish oil supplementation, between single-nucleotide polymorphisms (SNPs) within genes involved in fatty acid β-oxidation and dietary fat intakes. Two hundred and eight (208) participants were recruited in the greater Quebec City area. The participants completed a six-week fish oil supplementation (5 g fish oil/day: 1.9–2.2 g EPA and 1.1 g DHA). Dietary fat intakes were measured using three-day food records. SNPs within *RXRA*, *CPT1A*, *ACADVL*, *ACAA2*, *ABCD2*, *ACOX1* and *ACAA1* genes were genotyped using TAQMAN methodology. Gene-diet interaction effects on the plasma TG response were observed for SNPs within *RXRA* (rs11185660, rs10881576 and rs12339187) and *ACOX1* (rs17583163) genes. For rs11185660, fold changes in *RXRA* gene expression levels were different depending on SFA intakes for homozygotes T/T. Gene-diet interaction effects of SNPs within genes involved in fatty acid β-oxidation and dietary fat intakes may be important in understanding the inter-individual variability in plasma TG levels and in the plasma TG response to a fish oil supplementation.

## 1. Introduction

Plasma triglyceride (TG) level is an important risk factor for cardiovascular disease [1]. Twin studies have revealed that plasma TG levels are highly heritable (19%–72%) with additive genetic effects accounting for around 40% of the variability observed [2,3]. The environment also contributes for an important part of the variability observed. Proportions of macronutrient intake have an impact on plasma TG levels. For example, high-fat/low-carbohydrate isocaloric diets lead to a decrease in plasma TG levels compared to low-fat/high-carbohydrate diets [4]. Polyunsaturated fats (PUFA), especially *n*-3 PUFA, have been reported to have a beneficial impact on plasma TG levels [4,5]. At the opposite, saturated fat (SFA) intakes seem to increase intrahepatic TG levels and plasma TG levels [6,7]. Fabbrini *et al*. [8] have observed that very-low-density lipoprotein (VLDL) TG secretion was almost doubled among obese individuals with high intrahepatic TG levels.

Following the intake of *n*-3 PUFA supplements, an important inter-individual variability has been observed in the plasma TG response. Approximately 30% of the individuals do not lower their plasma TG levels [9,10,11]. It has been observed that fish oil intake reduces VLDL production with or without a concomitant increase in VLDL clearance [12]. An increase in fatty acid β-oxidation via an increase in *peroxisome proliferator-activated receptor alpha* (*PPARA*) gene expression induced by fish oil, may decrease fatty acid availability for VLDL production [12,13]. *PPARA* forms a heterodimer with *retinoid X receptor alpha* (*RXRA*) and regulates the activity of several genes involved in the fatty acid metabolism [14]. In mitochondrial fatty acid β-oxidation, *PPARA* regulates genes such as *carnitine palmitoyltransferase 1A* (*CPT1A*), *acyl-CoA dehydrogenase* (*ACADVL*) and *acetyl-CoA acyltransferase 2* (*ACAA2*) [14]. *PPARA* also regulates enzymes involved in peroxisomal β-oxidation such as *ATP-binding cassette, sub-family (ALD)*, *member 2* (*ABCD2*), *acyl-CoA oxidase 1* (*ACOX1*) and *acetyl-CoA acyltransferase 1* (*ACAA1*) [14,15,16]. Single-nucleotide polymorphisms (SNPs) within these genes may have an impact on the plasma TG lowering effects of fish oil. A few studies have observed associations with plasma TG levels, coronary heart disease risk and metabolic syndrome with SNPs located within *RXRA* gene [17,18,19]. A SNP within the *CPT1A* gene (rs80356779) was associated with high-density lipoprotein cholesterol (HDL-C) among Eskimos [20]. Interestingly, a gene-diet interaction effect on BMI was observed with the polymorphism pA275T of the *CPT1A* gene [21].

It is possible that gene-diet interaction effects modulate the plasma TG response to fish oil. The objective of this study is to examine the effects of SNPs within *RXRA*, *CPT1A*, *ACADVL*, *ACAA2*, *ABCD2*, *ACOX1* and *ACAA1* genes, dietary fat intakes and gene-diet interaction effects on the plasma TG response to fish oil. Gene-diet interaction effects with *RXRA* and *ACOX1* genes were observed on the plasma TG response to fish oil intake.

## 2. Experimental Section

### 2.1. Participants

A total of 254 subjects were recruited between September 2009 and December 2011 from the greater Quebec City metropolitan area through advertisements in local newspapers as well as by electronic messages sent to university students/employees. To be eligible, subjects had to be non-smokers and free of any thyroid or metabolic disorders requiring treatment such as diabetes, hypertension, severe dyslipidemia, and coronary heart disease. Participants had to be aged between 18 and 50 years with a BMI between 25 and 40 kg/m^2^. Subjects were excluded if they had taken *n*-3 PUFA supplements within 6 months prior to the study. A total of 210 subjects completed the *n*-3 PUFA supplementation period. However, TG levels were available for 208 participants, thus the analyses were conducted on 208 participants. The experimental protocol was approved by the ethics committees of Laval University Hospital Research Center and Laval University. This trial was registered at clinicaltrials.gov as NCT01343342.

### 2.2. Study Design and Diets

Subjects followed a run-in period of two weeks. Individual dietary instructions were given by a trained dietitian to achieve the recommendation from Canada’s Food Guide. Subjects were asked to follow these dietary recommendations and stably maintain their body weight throughout the protocol. Some specifications were given regarding the *n*-3 PUFA dietary intakes: not to exceed two fish or seafood servings per week, prefer white flesh fishes instead of fatty fishes (examples were given) and avoid enriched *n*-3 PUFA dietary products such as some milks, juices, breads and eggs. Subjects were also told to limit their alcohol consumption during the protocol; two regular drinks per week were allowed. In addition, subjects were not allowed to take *n*-3 PUFA supplements (such as flaxseed), vitamins or natural health products during the protocol.

After the 2-week run-in, each participant received a bottle containing the *n*-3 PUFA capsules for the next six weeks. They were instructed to take five (1 g oil each) capsules per day (Ocean Nutrition, Dartmouth, NS, Canada), providing a total of 5 g of fish oil (1.9–2.2 g EPA and 1.1 g DHA) per day. Capsules were provided in sufficient quantity for six weeks. Compliance was assessed from the return of bottles. Subjects were asked to report any deviation during the protocol and to write down their alcohol and fish consumption as well as any side effects. Before each phase, subjects received detailed written and oral instructions on their diet.

Subjects completed two 3-day food records (pre- and post-*n*-3 PUFA supplementation period). Dietary data included both foods and beverages consumed at home and outside. A dietitian provided instructions on how to complete the food record with some examples and a written copy of these examples. All foods and beverages consumed on two representative weekdays and one weekend day were weighed or estimated and recorded in food diaries. Dietary intake data were analyzed using Nutrition Data System for Research software version 2011 developed by the Nutrition Coordinating Center (NCC), University of Minnesota, Minneapolis, MN, USA.

### 2.3. Anthropometric Measurements

Body weight, height, and waist girth were measured according to the procedures recommended by the Airlie Conference [22] and were taken before the run-in period, as well as pre- and post- fish oil supplementation. BMI was calculated as weight per meter squared (kg/m^2^).

### 2.4. Biochemical Parameters

The morning after a 12-h overnight fast and 48-h alcohol abstinence, blood samples were collected from an antecubital vein into vacutainer tubes containing EDTA. Blood samples were used to identify individuals with metabolic disorders, which were excluded. Plasma was separated by centrifugation (2500× *g* for 10 min at 4 °C), samples were aliquoted and frozen for subsequent analyses. Plasma total cholesterol (TC) and TG were measured using enzymatic assays [23,24]. Infranatant (*d* > 1.006 g/mL) with heparin-manganese chloride was used to precipitate VLDL and low-density lipoprotein (LDL) and then determine HDL-C [25]. The equation of Friedewald was used to estimate LDL-cholesterol (LDL-C) levels [26]. Non-HDL-C was calculated by subtracting HDL-C from TC. Plasma apolipoprotein B-100 (apoB) concentrations were measured by the rocket immunoelectrophoretic method of Laurell, as previously described [27].

### 2.5. SNPs Selection and Genotyping

**Figure 1:**
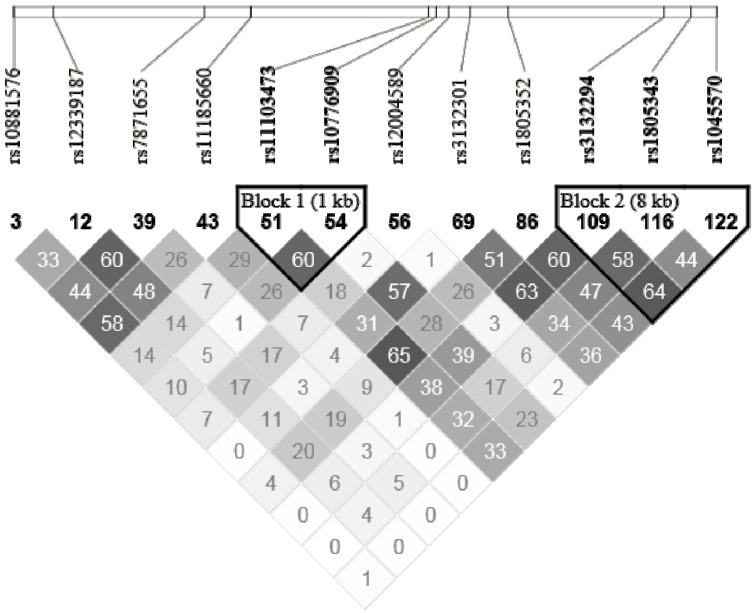
LD plot of *RXRA* gene.

**Figure 2:**
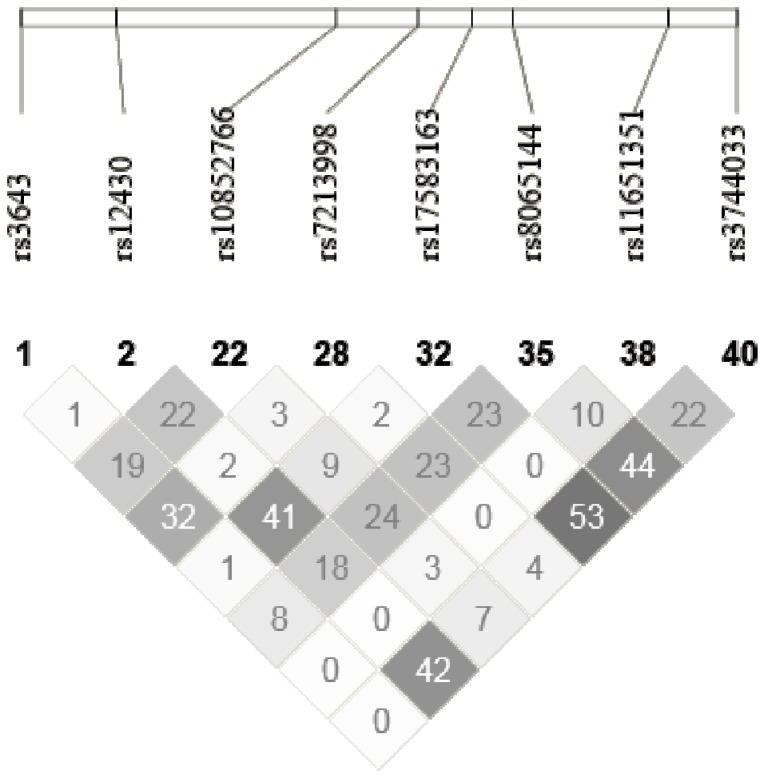
LD plot of *ACOX1* gene.

Genetic analyses were performed on genomic DNA isolated from human leukocytes. DNA was extracted from 200 μL of buffy coat using the GenElute™ Blood Genomic DNA Kit (Sigma-Aldrich, St. Louis, MO, USA). Spectrophotometric quantification was realised with NanoDrop 2000C UV-Vis Spectrophotometer (Thermo Scientific, Waltham, MA, USA). SNPs were selected with the International HapMap Project SNP database (HapMap Data Rel 28 Phase II + III, August 10, on National Center for Biotechnology Information (NCBI) B36 assembly, dbSNP b126). Tag SNPs (tSNPs) were determined with the tagger procedure in HaploView software version 4.2 (Broad Institute, Cambridge, MA, USA) with minor allele frequency (MAF) of >0.05 and pairwise tagging *R*^2^ ≥ 0.80. For each gene a minimum of 85% of the most common SNPs had to be captured by tSNPs. Additionally, tSNPs were prioritized according to the following criteria: (1) known SNPs from the literature; (2) SNPs within coding regions (exon); (3) SNPs within the promoter region (2500 bp before the start codon); (4) SNPs within 3′ UTR region (500 bp after the stop codon) and (5) SNPs within 100 bp before an exon-intron splicing boundaries. Afterwards, as shown in Figure 1 and Figure 2 and Supplementary Figures S1–S4, linkage disequilibrium (LD) plots were generated with Haploview software version 4.2. All tSNPs within *RXRA*, *CPT1A*, *ACADVL*, *ACAA2*, *ABCD2*, *ACOX1* and *ACAA1* genes were genotyped with the TAQMAN methodology [28], as described previously [11].

### 2.6. Gene Expression Assessment

Blood samples (pre- and post-supplementation) were collected into an 8-mL Cell Preparation Tube (CPT) (Becton Dickinson, Oakville, ON, Canada). Peripheral blood mononuclear cells (PBMCs) were separated by centrifugation (1500× *g*, 20 min, at room temperature) and washed according to the manufacturer’s instructions. Total RNA was extracted with RNeasy Plus Mini Kit (QIAGEN, Mississauga, ON, Canada) according to manufacturer’s protocol. Spectrophotometric quantification was realised with NanoDrop 2000C UV-Vis Spectrophotometer (Thermo Scientific, Waltham, MA, USA) and cDNA was generated using 400 ng of total RNA with the High Capacity cDNA Reverse Transcription Kit (Life Technologies™, Carlsbad, CA, USA). cDNA was mixed with TaqMan OpenArray^®^ Real-Time PCR Master Mix (#4462164, Life Technologies™, Carlsbad, CA, USA). The assays used were as follows: Hs01067636_m1 (NM_002957.4) (*RXRA*), Hs00912671_m1 (NM_001031847.2, NM_001876.3) (*CPT1A*), Hs00825606_g1 (NM_000018.3, NM_001033859.2, NM_001270447.1, NM_001270448.1) (*ACADVL*), Hs01557254_m1 (NM_006111.2) (*ACAA2*), Hs00193054_m1 (NM_005164.3) (*ABCD2*), Hs01074241_m1 (NM_001185039.1, NM_004035.6, NM_007292.5) (*ACOX1*) and Hs01576070_m1 (NM_001607.3, NR_024024.1) (*ACAA1*), and *GAPDH* Hs99999905_m1 as the housekeeping gene. All assays used the same fluorescent reporter probe (FAM dye labeled). All samples were run in triplicate on a QuantStudio™ 12K Flex Real-Time PCR (RT-PCR) System (Life Technologies™, Carlsbad, CA, USA) using 48-well plates TaqMan^®^ OpenArray^®^ RT PCR Inventoried Format 18 (Life Technologies™, Carlsbad, CA, USA). The RT-PCR results were analysed with ExpressionSuite software version 1.0.1 (Life Technologies™, Carlsbad, CA, USA).

### 2.7. Statistical Analyses

Hardy-Weinberg equilibrium was tested with the ALLELE procedure of SAS [29] version 9.3 using Fisher’s exact test (*p* < 0.01). When the genotype frequency for homozygotes of the minor allele was <5%, carriers (heterozygotes and homozygotes) of the minor allele were grouped in order to have appropriate statistical power.

Variables abnormally distributed were logarithmically transformed. Paired *t*-test were computed to detect differences in macronutrient intake (expressed as a proportion of energy intake) between pre- and post-supplementation values. To test the impact of dietary fat intake on the response of plasma TG to fish oil supplementation (relative difference: (post-supplementation plasma TG minus pre-supplementation plasma TG/pre-supplementation plasma TG) × 100), subjects were divided on the basis of fat intake (%), including the fish oil supplement using the median value as a cut-off point. Subjects having an intake lower or equal to the median value were considered as having “low” intakes and subjects having an intake higher than the median value were considered as having “high” intakes of dietary fat.

Differences in the plasma TG response were tested using analyses of variance with the GLM procedure in SAS and the type 3 sum of squares for unbalanced study design. To take into account the possible gene-diet interaction effects, the interaction term was added in the model (SNPxdietary fat intake) (adjusted for age, sex and BMI). In this model, dietary fat intakes were included as continuous variables. Statistical analyses related to relative gene expression levels were conducted using the 2^−∆∆CT^ for the gene expression response as described by Livak *et al*. [30,31], separately by genotype according to dietary fat intake group. Pearson correlations were performed to observe associations between the gene expression response and the plasma TG response. Since polymorphisms tested in complex diseases rarely account for a large amount of variance, characterized by very low *p*-values (*p* < 0.001), we decided to present the results before correction for multiple testing and using a *p*-value < 0.05. All statistical analyses were performed using SAS statistical software version 9.3 [29].

## 3. Results

### 3.1. Characteristics of the Study Population, Genetic Variants and Dietary Intakes

Descriptive characteristics of the study participants are shown in Table 1. All tSNPs were in Hardy-Weinberg equilibrium. The selected tSNPs from Haploview software are presented in Figure 1 and Figure 2 and supplementary material and the genetic information for each tSNP are shown in Table 2. For *RXRA* gene, 12 tSNPs covered 85% of the known genetic variability, for *CPT1A* gene, 9 tSNPs covered 85%, for *ACADVL* gene, 1 tSNP covered 100%, for *ACAA2* gene, 6 tSNPs covered 87%, for *ABCD2* gene, 8 tSNPs covered 85%, for *ACOX1* gene, 8 tSNPs covered 88% and for *ACAA1* gene, 3 tSNPs covered 83%.

Comparisons of dietary intakes pre- and post-supplementation are presented in Table 3. Briefly, proportions of the macronutrients remained mainly similar when considering dietary intakes expressed as a proportion of energy intake without taking into account *n*-3 PUFA supplements. However, when taking into account *n*-3 PUFA supplements, participants slightly decreased their carbohydrate intake (−1.9%) and SFA intakes (−0.8%) and increased their total fat (+2.7%) and PUFA intakes (+1.1%) (*p* = 0.0009, *p* = 0.001, *p* < 0.0001 and *p* < 0.0001, respectively). Protein and MUFA intakes remained similar (*p* = 0.12 and *p* = 0.65, respectively).

**Table 1:** Descriptive characteristics of the study cohort.

Variables	Men (*n* = 96)	Women (*n* = 112)	Means ± SD *
Age (years)	31.2 ± 8.1	30.5 ± 9.2	30.8 ± 8.7
BMI (kg/m^2^)	27.5 ± 3.6	28.2 ± 3.8	27.8 ± 3.7
Waist circumference (cm)	94.9 ± 11.0	92.0 ± 10.4	93.3 ± 10.8
Systolic blood pressure (mmHg)	118.09 ± 11.40	106.79 ± 13.29	112.03 ± 13.64
		(*n* = 111)	(*n* = 207)
Diastolic blood pressure (mmHg)	70.53 ± 9.13	68.68 ± 9.19	69.54 ± 9.19
		(*n* = 111)	(*n* = 207)
Fasting glucose (mmol/L)	5.09 ± 0.44	4.83 ± 0.56	4.95 ± 0.52
			(*n* = 208)
Fasting insulin (pmol/L)	79.50 ± 32.19	85.04 ± 38.20	82.51 ± 35.61
			(*n* = 206)
	(*n* = 94)		
Total-C (mmol/L)	4.80 ± 0.99	4.83 ± 1.02	4.82 ± 1.01
LDL-C (mmol/L)	2.91 ± 0.87	2.70 ± 0.86	2.79 ± 0.87
	(*n* = 95)		(*n* = 207)
HDL-C (mmol/L)	1.29 ± 0.31	1.61 ± 0.39	1.46 ± 0.39
Triglycerides (mmol/L)	1.32 ± 0.74	1.15 ± 0.53	1.23 ± 0.64
ApoB (g/L)	0.89 ± 0.25	0.84 ± 0.25	0.86 ± 0.25
	(*n* = 95)		(*n* = 207)

* Mean ± standard deviation (SD).

**Table 2:** The selected single-nucleotide polymorphisms within *RXRA*, *CPT1A*, *ACADVL*, *ABCD2*, *ACOX1* and *ACAA1* genes.

Genes	dbSNP No. ^1^	Sequence ^2^	Position	MAF	Genotype Frequency		
*RXRA*	rs10881576	GCGGGTG[C/T]GGACCGC	Intron	0.28	C/C	C/T	T/T
					(*n* = 106)	(*n* = 86)	(*n* = 16)
					0.51	0.414	0.077
	rs7871655	CAGAATT[C/G]CGGGTGA	Intron	0.26	G/G	C/G	C/C
					(*n* = 110)	(*n* = 87)	(*n* = 11)
					0.529	0.418	0.053
	rs12339187	GGACCAG[A/G]TGTTTTA	Intron	0.17	A/A	A/G	G/G
					(*n* = 143)	(*n* = 60)	(*n* = 5)
					0.688	0.289	0.024
	rs11185660	CTGTGTC[C/T]CTGGAGA	Intron	0.27	T/T	C/T	C/C
					(*n* = 109)	(*n* = 87)	(*n* = 12)
					0.524	0.418	0.058
	rs11103473	TCTCTCC[A/T]AACTATT	Intron	0.36	A/A	A/T	T/T
					(*n* = 81)	(*n* = 105)	(*n* = 22)
					0.389	0.505	0.106
	rs10776909	GTGGGGA[C/T]TTTGAGT	Intron	0.23	C/C	C/T	T/T
					(*n* = 120)	(*n* = 80)	(*n* = 8)
					0.577	0.385	0.039
	rs12004589	GCTCCCT[G/T]CATGGCC	Intron	0.08	G/G	G/T	T/T
					(*n* = 178)	(*n* = 28)	(*n* = 2)
					0.856	0.135	0.01
	rs3132301	TGCTGAG[C/T]CCCCCAG	Intron	0.22	C/C	C/T	T/T
					(*n* = 125)	(*n* = 76)	(*n* = 7)
					0.601	0.365	0.034
	rs1805352	ATAGGGA[A/C]AAACCTG	Intron	0.31	A/A	A/C	C/C
					(*n* = 97)	(*n* = 95)	(*n* = 16)
					0.466	0.457	0.077
	rs3132294	GAACACT[A/G]TGAACCG	Intron	0.23	G/G	A/G	A/A
					(*n* = 121)	(*n* = 77)	(*n* = 10)
					0.582	0.37	0.048
	rs1805343	CTTGCCC[A/G]GCCCTCA	Intron	0.37	A/A	A/G	G/G
					(*n* = 85)	(*n* = 93)	(*n* = 30)
					0.409	0.447	0.144
	rs1045570	CGTGGCC[G/T]CAGGTGC	3′UTR	0.16	G/G	G/T	T/T
					(*n* = 146)	(*n* = 57)	(*n* = 5)
					0.702	0.274	0.024
*CPT1A*	rs3019598	GTGCCCC[C/T]GTTACCT	Intron	0.35	C/C	C/T	T/T
					(*n* = 88)	(*n* = 93)	(*n* = 27)
					0.423	0.447	0.13
	rs897048	GCTGTCA[C/G]ACCGGGC	Intron	0.19	C/C	C/G	G/G
					(*n* = 134)	(*n* = 68)	(*n* = 6)
					0.644	0.327	0.029
	rs7942147	GGACACC[A/C]TGTGGCA	Intron	0.16	C/C	A/C	A/A
					(*n* = 144)	(*n* = 60)	(*n* = 4)
					0.692	0.289	0.019
	rs4930248	TCAGGGT[C/T]GCTTTGG	Intron	0.44	T/T	C/T	C/C
					(*n* = 62)	(*n* = 108)	(*n* = 38)
					0.298	0.519	0.183
	rs11228364	CTTCGAG[C/T]GCAGATC	Intron	0.1	C/C	C/T	T/T
					(*n* = 169)	(*n* = 36)	(*n* = 3)
					0.813	0.173	0.014
	rs11228368	CCAGAAG[A/G]GGGCACA	Intron	0.5	G/G	A/G	A/A
					(*n* = 52)	(*n* = 105)	(*n* = 51)
					0.25	0.505	0.245
	rs10896371	CTCGTTC[C/T]CACAAAT	Intron	0.14	T/T	C/T	C/C
					(*n* = 153)	(*n* = 51)	(*n* = 4)
					0.736	0.245	0.019
	rs1017640	CTGGCCA[C/T]GTAATCA	Intron	0.1	C/C	C/T	T/T
					(*n* = 169)	(*n* = 37)	(*n* = 2)
					0.813	0.178	0.01
	rs613084	TTCAGTG[A/C]CACACCC	Intron	0.35	C/C	A/C	A/A
					(*n* = 89)	(*n* = 93)	(*n* = 26)
					0.428	0.447	0.125
*ACADVL*	rs2017365	GGCACAT[A/G]GTCTCTG	NearGene-5	0.38	A/A	A/G	G/G
					(*n* = 81)	(*n* = 96)	(*n* = 31)
					0.389	0.462	0.149
*ACAA2*	rs529556	ACTTTTT[C/T]AGGACTC	Intron	0.43	T/T	C/T	C/C
					(*n* = 76)	(*n* = 85)	(*n* = 47)
					0.365	0.409	0.226
	rs10502901	AAGCTAA[A/T]CTGTGTG	Intron	0.06	T/T	A/T	A/A
					(*n* = 184)	(*n* = 24)	(*n* = 0)
					0.885	0.115	0
	rs631536	ATTGACT[A/G]TGGTTAC	Intron	0.14	A/A	A/G	G/G
					(*n* = 150)	(*n* = 56)	(*n* = 2)
					0.721	0.269	0.01
	rs1942421	CTGTTCT[C/T]TCTTAAC	Intron	0.36	C/C	C/T	T/T
					(*n* = 91)	(*n* = 84)	(*n* = 33)
					0.438	0.404	0.159
	rs2276168	AGTATCA[A/T]CACAAGG	Intron	0.23	A/A	A/T	T/T
					(*n* = 128)	(*n* = 66)	(*n* = 14)
					0.615	0.317	0.067
	rs7237253	CCTTATA[A/G]TCATATT	3′UTR	0.1	A/A	A/G	G/G
					(*n* = 170)	(*n* = 36)	(*n* = 2)
					0.817	0.173	0.01
*ABCD2*	rs4072006	GAGAATG[A/G]CTAGAGG	NearGene-5	0.13	G/G	A/G	A/G
					(*n* = 159)	(*n* = 46)	(*n* = 3)
					0.764	0.221	0.014
	rs10877201	CTATAAT[C/T]CTTTAAC	Intron	0.2	C/C	C/T	T/T
					(*n* = 132)	(*n* = 68)	(*n* = 8)
					0.635	0.327	0.039
	rs12582802	GAGGTTT[A/G]TTTCCAA	Intron	0.06	A/A	A/G	G/G
					(*n* = 186)	(*n* = 21)	(*n* = 1)
					0.894	0.101	0.005
	rs4294600	ACTAAAT[A/G]TCACTCA	3′UTR	0.12	G/G	A/G	A/A
					(*n* = 161)	(*n* = 44)	(*n* = 3)
					0.774	0.212	0.014
	rs11172696	AGGGAAA[C/T]ATTGTAT	Intron	0.08	T/T	C/T	C/C
					(*n* = 177)	(*n* = 29)	(*n* = 2)
					0.851	0.139	0.01
	rs10877173	ACAAGTT[C/T]GGCTTTT	Intron	0.07	T/T	C/T	C/C
					(*n* = 184)	(*n* = 21)	(*n* = 3)
					0.885	0.101	0.014
	rs7133376	GGTAAAG[A/G]TGTATGA	Intron	0.2	G/G	A/G	A/A
					(*n* = 132)	(*n* = 67)	(*n* = 9)
					0.635	0.322	0.043
	rs7968837	ATATTAA[A/C]TTTACCA	3′UTR	0.27	C/C	A/C	A/A
					(*n* = 109)	(*n* = 86)	(*n* = 13)
					0.524	0.414	0.063
*ACOX1*	rs10852766	AAGAAAG[C/T]GCTCAGT	Intron	0.43	C/C	C/T	T/T
					(*n* = 74)	(*n* = 90)	(*n* = 44)
					0.356	0.433	0.212
	rs3744033	GCCTTCA[A/G]GGAGAAG	Intron	0.17	A/A	A/G	G/G
					(*n* = 142)	(*n* = 60)	(*n* = 6)
					0.683	0.289	0.029
	rs12430	TCCCAGA[C/T]GTAGCAC	3′UTR	0.11	C/C	C/T	T/T
					(*n* = 165)	(*n* = 39)	(*n* = 4)
					0.793	0.188	0.019
	rs8065144	AAGCCTC[A/G]AAAATGG	Intron	0.36	A/A	A/G	G/G
					(*n* = 89)	(*n* = 90)	(*n* = 29)
					0.428	0.433	0.139
	rs11651351	CTATTGC[C/T]GATCTCC	Intron	0.05	C/C	C/T	T/T
					(*n* = 188)	(*n* = 20)	(*n* = 0)
					0.904	0.096	0
	rs3643	GTAGTTT[C/T]GCTTACC	3′UTR	0.12	T/T	C/T	C/C
					(*n* = 166)	(*n* = 36)	(*n* = 6)
					0.798	0.173	0.029
	rs7213998	TCTGAAA[C/T]GTCAGAG	Intron	0.11	C/C	C/T	T/T
					(*n* = 169)	(*n* = 34)	(*n* = 5)
					0.813	0.164	0.024
	rs17583163	GATTTCC[C/T]CTGATGA	Intron	0.08	T/T	C/T	C/C
					(*n* = 176)	(*n*n = 1)
					0.846	0.149	0.005
*ACAA1*	rs5875	TACCATG[A/T]CATCAGT	3′UTR	0.14	T/T	A/T	A/A
					(*n* = 155)	(*n* = 48)	(*n* = 5)
					0.745	0.231	0.024
	rs2239621	CCTTCTA[C/T]TCCTATG	Intron	0.32	C/C	C/T	T/T
					(*n* = 97)	(*n* = 90)	(*n* = 21)
					0.466	0.433	0.101
	rs156265	TGGCCTT[C/G]TCCTTCT	Exon (missense Glu→Asp)	0.16	C/C	C/G	G/G
					(*n* = 149)	(*n* = 53)	(*n* = 6)
					0.716	0.255	0.029

^1^ SNP reference id from dbSNP Short Genetic Variations NCBI Reference Assembly; ^2^ Gene sequence from dbSNP Short Genetic Variations NCBI Reference Assembly.

**Table 3:** Dietary intakes pre-supplementation and post-supplementation.

Dietary Intakes	Pre-Supplementation (*n* = 207)	Post-Supplementation (*n* = 208)		*p*-Value (Without *n*-3 PUFA) *	*p*-Value (With *n*-3 PUFA) *
	Without *n*-3 PUFA Supplements	Without *n*-3 PUFA Supplements	With *n*-3 PUFA Supplements		
Energy (kcal)	2273 ± 590	2144 ± 566	2186 ± 566	<0.0001	0.006
Carbohydrate (%)	50.5 ± 7.2	49.4 ± 7.7	48.6 ± 7.8	<0.05	0.0009
Protein (%)	17.4 ± 3.3 (*n* = 206)	17.5 ± 3.4	17.0 ± 3.2	0.66	0.12
Total fat (%)	32.6 ± 6.0	33.3 ± 6.4	35.3 ± 6.3	0.15	<0.0001
SFA (%)	11.2 ± 3.6	11.5 ± 3.3	10.4 ± 3.0	0.13	0.001
MUFA (%)	11.9 ± 2.8	12.0 ± 3.2	12.0 ± 3.3	0.45	0.65
PUFA (%)	5.9 ± 2.0	5.8 ± 2.1	7.0 ± 2.1	0.56	<0.0001

* *p*-Values provided by a paired *t*-test.

### 3.2. Associations between Dietary Fat Intakes and the Plasma TG Response

When observing the impact of dietary fat consumption on the plasma TG response (%) to a fish oil supplementation, a trend was observed only for SFA intake (*p* = 0.08) (adjusted for age, sex and BMI) (high SFA > 10.48% and low SFA ≤ 10.48%). Individuals with high SFA intakes had a smaller relative decrease in plasma TG levels following the intake of fish oil than individuals with low SFA intake (−8.77% ± 25.56% compared to −15.01% ± 26.01%). No significant differences were observed between SFA intake groups and baseline plasma TG levels (*p* = 0.76).

### 3.3. Associations between tSNPs, Relative Gene Expression Levels and the Plasma TG Response Following Fish Oil Supplementation

None of the tSNPs were associated with the plasma TG response. When observing the fold change gene expression in response to the fish oil supplementation (using the 2^−∆∆CT^), fold change in gene expression levels of *CPT1A* were inversely related to the relative delta TG (*r* = −0.15, *p* = 0.03). These results suggest that a greater increase in *CPT1A* gene expression was associated with a more important reduction in plasma TG following the intake of fish oil. No relationships were observed for the other genes (data not shown).

### 3.4. Gene-Diet Interaction Effects on the Plasma TG Response and on the Gene Expression Response Following Fish Oil Supplementation

**Figure 3:**
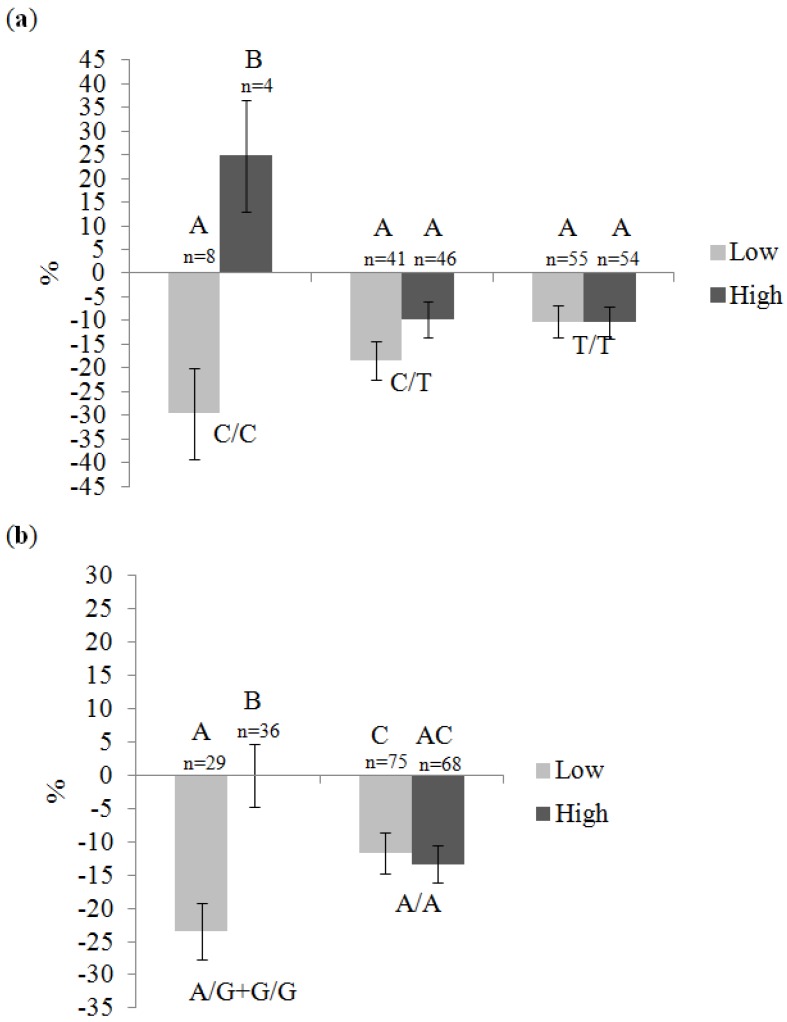
The plasma TG response following fish oil intake according to genotype of *RXRA* gene and dietary fat intakes (Means ± SE). Means with different letters are significantly different (assessed by an ANOVA). Dietary fats are separated according to the median value (Low or High). (**a**) rs11185660 and saturated fat intakes (≤10.48% or >10.48%), and (**b**) rs12339187 and saturated fat intakes (≤10.48% or >10.48%).

Significant gene-diet interaction effects on the plasma TG response are presented in Table 4. Briefly, one tSNP (rs11185660) within *RXRA* gene interacted with total fat intakes, three tSNPs (rs10881576, rs12339187 and rs11185660) within *RXRA* gene interacted with SFA intakes and one tSNP (rs17583163) within *ACOX1* gene interacted with PUFA intakes to affect the plasma TG response. Figure 3 illustrates the interaction effect on the plasma TG response according to genotype and dietary fat intake group (low or high). Briefly, for the tSNP rs11185660, C/C homozygotes with high SFA intakes (a trend was also observed for total fat intakes) increased their plasma TG levels following the intake of fish oil whereas those with low SFA intakes decreased their plasma TG levels. Among C/T heterozygotes and T/T homozygotes, the decrease in plasma TG levels following the *n*-3 PUFA supplementation was comparable with either a high or a low SFA intake. For the tSNP rs12339187, carriers of the minor G allele with low SFA intakes had a greater decrease in plasma TG levels compared to carriers of the G allele with high SFA intakes or to A/A homozygotes with high or low SFA intakes.

**Table 4:** Gene-diet interaction effects on the plasma TG response.

Gene	tSNP	Genotype	β (Interaction Term) ^1^	*P* Genotype ^2^	*P* Dietary Fat Intake ^2^	*P* Interaction Effect ^2^
Total fat intakes (%)						
*RXRA*	rs11185660	C/C	3.70 ± 1.16	0.004	0.0009	0.004
		C/T	1.02 ± 0.61			
		T/T	0			
Saturated fat intakes (%)						
*RXRA*	rs10881576	T/T	8.52 ± 2.61	0.007	0.0004	0.004
		C/T	1.84 ± 1.21			
		C/C	0			
	rs12339187	A/G + G/G	3.20 ± 1.25	0.02	0.005	0.01
		A/A	0			
	rs11185660	C/C	9.39 ± 2.66	0.003	<0.0001	0.002
		C/T	1.69 ± 1.23			
		T/T	0			
Polyunsaturated fat intakes (%)						
*ACOX1*	rs17583163	C/C + C/T	6.79 ± 2.34	0.02	0.09	0.004
		T/T	0			

^1^ Homozygotes for the major allele is the reference group; ^2^ *p*-values were determined with an ANOVA using dietary fat intakes as continuous values adjusted for age, sex and BMI.

None of the gene interaction effect had an impact on gene expression response following the fish oil supplementation. However, when participants were first stratified on the basis of dietary fat intakes and then on the basis of the genotype a few differences were observed. As shown in Table 5, the fold change in *RXRA* gene expression levels due to the fish oil supplementation was different for T/T homozygotes of rs11185660. For this genotype, individuals with high total fat intakes had a mean fold change due to the fish oil supplementation of −1.08 (post-compared to pre-supplementation) compared to a mean fold change of 1.05 for individuals with low total fat intakes (*p* = 0.01). No significant differences were observed for C/C and C/T genotypes (*p* = 0.52 and *p* = 0.86, respectively). For the tSNP rs12339187, a trend was observed for A/A homozygotes (*p* = 0.06). For these individuals, when SFA intakes were high, *RXRA* gene expression levels slightly decreased with the fish oil supplementation and increased with low SFA intakes.

**Table 5:** Gene expression response according to dietary fat intake and genotype.

Gene	SNP	Genotype	Total Fat Intake ^1^		*p* ^2^
			Low (≤35.23%)	High (>35.23%)	
*RXRA*	rs11185660	C/C	1.12-fold (*n* = 7)	−1.14-fold (*n* = 5)	0.52
		C/T	−1.05-fold (*n* = 43)	−1.04-fold (*n* = 44)	0.86
		T/T	1.05-fold (*n* = 54)	−1.08-fold (*n* = 53)	0.01
**Gene**	**SNP**	**Genotype**	**Saturated Fat Intake** ^1^		***p*** ^2^
			**Low** **(≤10.48%)**	**High** **(>10.48%)**	
*RXRA*	rs12339187	A/G + G/G	−1.07-fold (*n* = 29)	−1.05-fold (*n* = 36)	0.92
		A/A	1.04-fold (*n* = 74)	−1.06-fold (*n* = 67)	0.06

^1^ The fold change represents post-supplementation relative gene expression levels compared to pre-supplementation relative gene expression levels. Fold change = 2^−∆∆CT^ = 2^−(post-supplementation ∆CT-pre-supplementation ∆CT)^; ^2^ *p* values were calculated with an ANOVA adjusted for age, sex and BMI.

## 4. Discussion

Macronutrient intakes before the fish oil supplementation were comparable to intakes reported among Canada’s population [32,33]. During this study, macronutrient intakes remained mostly constant. However, participants spontaneously reduced their energy intakes which may be related to the reduction observed in the intake of carbohydrates. The intake of DHA has been associated with reductions in energy intake among free living healthy men [34]. This effect was explained by an increase in the release of appetite hormone cholecystokinin [34]. This spontaneous reduction in energy intakes could also be caused by the Hawthorne effect, which involves that participants reduced their energy intakes only by knowing that they were in a study [35].

Among factors modulating the plasma TG response to fish oil intake, SFA intakes may be an important factor. The relative decrease in plasma TG levels of individuals with high SFA intakes was almost reduced by half compared to individuals with low SFA intakes (−9% compared to −15%). Moreover, there were no differences in baseline plasma TG levels between SFA intake groups. As mentioned previously, high SFA intakes increase intrahepatic TG levels which could enhance hepatic VLDL-TG secretion [6,7,8]. Contrary to PUFA, SFA increases the activity of *hepatic nuclear factor 4-α* (*HNF4A*) which is a transcription factor acting as a homodimer to activate several hepatic genes encoding apolipoproteins, including apoB [36,37]. It has been also observed that a diet high in SFA increases plasma TG levels possibly via an increase in apolipoprotein C-III gene (*APOC3*) mediated by *HNF4A* gene [37]. Globally, the impacts of SFA on the regulation of lipid metabolism could partly counteract the plasma TG lowering effects of *n*-3 PUFA which could lead to smaller decreases after the intake of fish oil, as observed in this study.

Following fish oil intake, only the genetic variability within *RXRA* and *ACOX1* genes seemed to be associated with differences in the plasma TG response (using the relative difference in plasma TG between post-supplementation and pre-supplementation). The impact of the presence of these intronic SNPs is unknown. However, gene expression levels tended to be different according to the genotype for two of these SNPs depending on dietary fat intakes. Intronic SNPs could also be in LD with other functional SNPs or depending on splicing events, some of these SNPs may be in translated regions. The modulation of the activity of genes or encoded enzymes related to fatty acid β-oxidation may modulate fatty acid availability for VLDL-TG hepatic production, therefore contributing to modulate plasma TG levels [7,38,39]. The protein encoded by *RXRA* gene forms a heterodimer with PPARA transcription factor and affects the expression of many genes involved in fatty acid β-oxidation [40]. Fatty acids have been shown to be natural PPARA ligands [41]. Both SFA and unsaturated fatty acids are able to bind with PPARA, but long-chain *n*-3 PUFAs seem to be the most potent activators [41]. Moreover, RXRA may bind other transcription factors or act as a homodimer on other pathways, which could also have an impact on lipid metabolism [40]. A few studies have reported associations with SNPs or haplotypes within *RXRA* gene with plasma TG levels or the metabolic syndrome [17,19]. In this study, a few SNPs within *RXRA* gene interacted with dietary fats and were associated with the plasma TG response following fish oil intake (rs11185660, rs10881576 and rs12339187). Peloso *et al*. [18] have observed a decreased risk of having low HDL-C and coronary artery disease among carriers of the minor C allele compared to homozygotes T/T of rs11185660. However, dietary fat intakes were not taken into account. In this study, the T/T genotype of rs11185660 depending on dietary fat intakes was also associated with differences in gene expression levels following fish oil intake. *ACOX1* gene encodes for the first enzyme in peroxisomal fatty acid β-oxidation and is regulated by the PPARA transcription factor [42]. To our knowledge, none of the SNPs within the *ACOX1* gene have been studied in the context of lipid metabolism. One gene-diet interaction effect on the plasma TG response was observed with rs17583163 and PUFA intakes. Dietary fish oil has been shown to induce *ACOX1* gene expression in the liver, skeletal muscle and heart [43] which was not observed in this study (data not shown). Various transcripts have been reported for this gene that are likely to be differently regulated by PUFA intakes and possibly explaining the lack of association in the present study with an assay targeting three transcripts. Alternatively, the impact of dietary fish oil may be dependent on the intake of other dietary lipids and genetic variants.

The *CPT1A* gene encodes for an essential transporter required for the initiation of fatty acid β-oxidation in the mitochondria [44]. In this study, a greater increase in *CPT1A* relative gene expression levels following the intake of fish oil was associated with a more important reduction in plasma TG levels. *CPT1A* gene expression levels have been previously reported to increase following the intake of *n*-3 PUFA [45,46]. Radler *et al*. [46] have observed that the intake of a yogurt composed of *n*-3 PUFA, polyphenols and l-carnitine induced *CPT1A* gene expression among overweight moderately hyperlipidemic individuals. However, in this study mean *CPT1A* gene expression levels remained unchanged following the intake of fish oil (data not shown). This may be attributable to the healthy status of the study participants which could lead to too subtle differences in gene expression levels to be detected.

In this study, the presence of certain SNPs within genes involved in fatty acid β-oxidation depending on dietary fat intakes modulated the plasma TG response following fish oil intake. Because of the potentially small impacts of the gene-diet interaction effects among healthy individuals on the plasma TG response following fish oil, data was shown before correction for multiple testing. However, this could lead to false positive results. Thus, these results need to be replicated in order to properly determine relevant gene-diet interaction effects.

## 5. Conclusions

Globally, gene-diet interaction effects with *RXRA* and *ACOX1* genes were observed on the plasma TG response to fish oil intake. An increase in *CPT1A* gene expression was associated with a more important decrease in plasma TG levels. Moreover, higher SFA intakes tended to decrease the plasma TG response to fish oil. In conclusion, these results indicate that gene-diet interaction effects may modulate the response of plasma TG levels to fish oil intake, and contribute to the explanation of the inter-individual variability observed.

## Supplementary Files

Supplementary File 1 Supplementary Information (DOCX, 91 KB)
