# Supplementary material for: Polymorphisms in Genes Involved in Fatty Acid β-Oxidation Interact with Dietary Fat Intakes to Modulate the Plasma TG Response to a Fish Oil Supplementation
Source: Nutrients. 2014 Mar 18;6(3):1145–63. doi: 10.3390/nu6031145 (PMC3967183; doi:10.3390/nu6031145)
Supplement: Supplementary File 1 — Supplementary Information (DOCX, 91 KB) [file nutrients-06-01145-s001.docx]

**Supplementary Information**

**Figure S1.** LD plot of *CPT1A* gene.


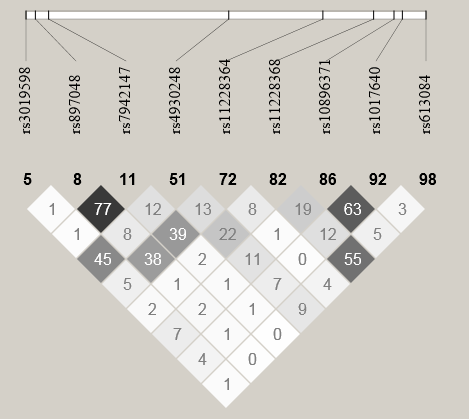


**Figure S2.** LD plot of *ACAA2* gene.


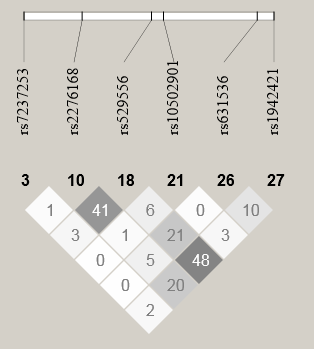


**Figure S3.** LD plot of *ABCD2* gene.


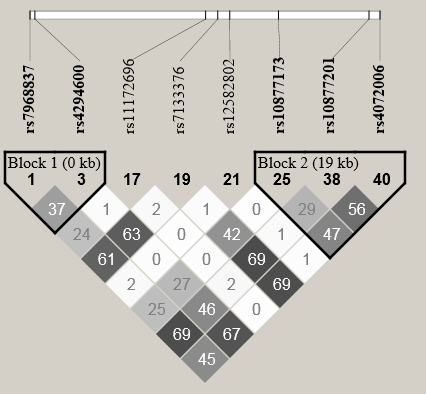


**Figure S4.** LD plot of *ACAA1* gene.


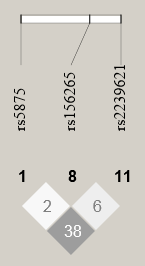


© 2014 by the authors; licensee MDPI, Basel, Switzerland. This article is an open access article distributed under the terms and conditions of the Creative Commons Attribution license (http://creativecommons.org/licenses/by/3.0/).
